# Associations between advanced cancer patients' survival and family caregiver presence and burden

**DOI:** 10.1002/cam4.653

**Published:** 2016-02-10

**Authors:** J. Nicholas Dionne‐Odom, Jay G. Hull, Michelle Y. Martin, Kathleen Doyle Lyons, Anna T. Prescott, Tor Tosteson, Zhongze Li, Imatullah Akyar, Dheeraj Raju, Marie A. Bakitas

**Affiliations:** ^1^ School of NursingUniversity of Alabama at BirminghamBirminghamAlabama; ^2^ Departments of Psychological and Brain SciencesDartmouth CollegeHanoverNew Hampshire; ^3^ Division of Preventive MedicineUniversity of Alabama at BirminghamBirminghamAlabama; ^4^ Department of Preventive MedicineUniversity of Tennessee Health Science CenteMemphisTennessee; ^5^ Department of PsychiatryGeisel School of Medicine at DartmouthHanoverNew Hampshire; ^6^ Biostatistics Shared ResourceNorris Cotton Cancer CenterLebanonNew Hampshire; ^7^ School of NursingHacettepe UniversityAnkaraTurkey; ^8^ Department of MedicineDivision of GerontologyGeriatrics, and Palliative CareUniversity of Alabama at BirminghamBirminghamAlabama

**Keywords:** Advanced cancer, family caregivers, patient survival

## Abstract

We conducted a randomized controlled trial (RCT) of an early palliative care intervention (ENABLE: Educate, Nurture, Advise, Before Life Ends) for persons with advanced cancer and their family caregivers. Not all patient participants had a caregiver coparticipant; hence, we explored whether there were relationships between patient survival, having an enrolled caregiver, and caregiver outcomes prior to death. One hundred and twenty‐three patient‐caregiver dyads and 84 patients without a caregiver coparticipant participated in the ENABLE early versus delayed (12 weeks later) RCT. We collected caregiver quality‐of‐life (QOL), depression, and burden (objective, stress, and demand) measures every 6 weeks for 24 weeks and every 3 months thereafter until the patient's death or study completion. We conducted survival analyses using log‐rank and Cox proportional hazards models. Patients with a caregiver coparticipant had significantly shorter survival (Wald = 4.31, HR = 1.52, CI: 1.02–2.25, *P *= 0.04). After including caregiver status, marital status (married/unmarried), their interaction, and relevant covariates, caregiver status (Wald = 6.25, HR = 2.62, CI: 1.23–5.59, *P *= 0.01), being married (Wald = 8.79, HR = 2.92, CI: 1.44–5.91, *P* = 0.003), and their interaction (Wald = 5.18, HR = 0.35, CI: 0.14–0.87, *P* = 0.02) were significant predictors of lower patient survival. Lower survival in patients with a caregiver was significantly related to higher caregiver demand burden (Wald = 4.87, CI: 1.01–1.20, *P* = 0.03) but not caregiver QOL, depression, and objective and stress burden. Advanced cancer patients with caregivers enrolled in a clinical trial had lower survival than patients without caregivers; however, this mortality risk was mostly attributable to higher survival by unmarried patients without caregivers. Higher caregiver demand burden was also associated with decreased patient survival.

## Introduction

We conducted a fast track randomized controlled trial (RCT) with 207 newly diagnosed advanced cancer patients and 123 family caregivers to test the ENABLE III (Educate, Nurture, Advise, Before Life Ends) nurse‐led, telephone‐based palliative care intervention. This RCT demonstrated that concurrent palliative care initiated soon after a diagnosis of advanced cancer had beneficial effects on patient survival and caregiver depression and burden compared with initiating this intervention 12 weeks later 1, 2. Because we enrolled patient participants with and without family caregivers, we had a “natural” experiment 3 allowing us to examine whether the presence of a caregiver and a caregiver's wellbeing and burden might also be related to patient survival.

Caregivers perform a plethora of health‐related tasks that appear vital for advanced cancer patients' survival. They spend an average of 8 h/day 4 tracking and treating symptoms; administering medications and breathing treatments; coordinating medical appointments; participating in advance care planning and other healthcare decision‐making; providing emotional and spiritual support; preparing meals; managing finances; and performing domestic home duties 5, 6, 7, 8. Over time, however, caregivers may become burdened by performing these tasks.

Part of this burden relates to caregivers feeling untrained and unprepared 6, 8, 9, while other parts relate to witnessing someone close to them struggle with serious illness. Thus, caregivers become prone to depression 10, anxiety 11, and poor physical health 12, 13. As the Institute of Medicine 14 and others 15, 16 have reported, this poor caregiver health can attenuate a caregiver's ability to provide care to cancer patients. This line of reasoning served as the impetus for us to include a parallel intervention specifically for family caregivers in the ENABLE III trial. Nurse coaches provided one‐on‐one support and education to caregivers about caregiving tasks, and about how to cope with their care recipients' struggle with serious illness. We believed this would improve caregivers' wellbeing and performance thus benefiting cancer patients' wellbeing and survival. Indeed, the main results of our trial demonstrated that caregivers had lower depressed mood and stress burden 2.

However, two unanswered questions remained: (1) What relationship exists between having a family caregiver and a patient's survival? (2) What relationships exist between a caregiver's outcomes (quality‐of‐life [QOL], depression, and burden) and a patient's survival? To address these questions, we conducted a secondary analysis of our clinical trial data. We hypothesized that having a caregiver would be associated with increased patient survival, and that higher caregiver QOL and lower caregiver depression and burden would be associated with increased patient survival.

## Methods

This was a secondary analysis of the ENABLE III 1, 2 “fast track” RCT 17. Individuals with newly diagnosed, recurrent, or progressive metastatic cancer and their caregivers (if they had one and were willing to enroll) were randomly assigned to receive the intervention as soon as possible after diagnosis (early group) or 12 weeks later (delayed group). The study protocol, data and safety monitoring plan were approved by the Norris Cotton Cancer Center/Dartmouth College and the Veterans Administration (VA) Medical Center, White River Junction, Vermont institutional review boards. The trial was registered in clinicaltrials.gov (Identifier NCT01245621).

### Sample and setting

Patient and caregiver participants in the ENABLE III trial were recruited between October 11, 2010 and March 5, 2013 from the Norris Cotton Cancer Center at Dartmouth‐Hitchcock Medical Center (DHMC), affiliated DHMC outreach clinics, and the White River Junction, Vermont VA Medical Center. Patient inclusion criteria were: (1) age >18 years; (2) within 30–60 days of being informed by a treating oncologist of a new diagnosis, recurrence, or progression of an advanced‐stage cancer; (3) oncologist‐estimated prognosis of 6–24 months; (4) English speaking; and (5) able to complete baseline questionnaires. Patients were excluded if: they scored <4 on the Callahan Cognitive Screen; 18 had an active Axis I psychiatric condition (e.g., schizophrenia, bipolar disorder) or substance use disorder; or had uncorrectable hearing disorder or unreliable telephone service. Patient participants were asked to nominate a family caregiver, defined as “a person who knows you well and is involved in your medical care” to participate in a parallel intervention. Patients were not excluded if they did not have a participating caregiver. There were no other caregiver eligibility criteria. After completing baseline measures, patients and their caregiver coparticipants, if they had one, were randomly assigned to receive early or delayed intervention.

### The ENABLE III early palliative care intervention

The ENABLE III intervention was initially developed in 1998 as a Robert Wood Johnson demonstration project to integrate early palliative care with oncology care and has now been refined and evaluated in two large multisite RCTs 1, 2, 19, 20, 21. Details of the intervention are described elsewhere 1, 2, 20 and on the National Cancer Institute's Research‐tested Intervention Programs website (rtips.cancer.gov/rtips/index.do). Briefly, the ENABLE III intervention consisted of: (1) an outpatient palliative care assessment following National Consensus Guidelines 22 (caregivers invited to attend); (2) a series of individualized phone sessions delivered weekly by nurse coaches following a guidebook called Charting Your Course (patients: 6 sessions; caregivers: 3 sessions); and (3) monthly follow‐up to reinforce previous content as needed or to address new issues. Patients and caregivers received their one‐on‐one sessions with a separate nurse coach. The first three patient and caregiver sessions addressed decision‐making and problem‐solving strategies (based upon the principles of Problem‐solving Treatment and the COPE program) 23, 24, 25, 26, communication skills, advance care planning, and symptom management. The patient portion of ENABLE III included three more sessions that incorporated the Outlook life review intervention developed by Steinhauser and colleagues 27, 28.

### Data collection and measures

Caregiver outcome measures were collected by telephone by a study coordinator blinded to group assignment at baseline and every 6 weeks for 24 weeks and then every 12 weeks until the patient's death or study completion. Caregiver QOL was measured using the 35‐item Caregiver QOL‐Cancer Scale (CQOL‐C) (score range 0–140: higher scores = worse QOL) 29. Domains of the CQOL‐C include physical, emotional, and spiritual wellbeing related to caregiving and relationship quality with the care recipient. Internal consistency has been reported as 0.91 and a test–retest reliability as 0.95. Caregiver depressed mood was measured using the 20‐item Center for Epidemiological Studies Depression (CES‐D) scale (score range 0–60; higher scores = higher depressed mood; >16 = clinically significant depression) 30, 31. Caregiver burden was measured using the 14‐item Montgomery Borgatta Caregiver Burden Scale (MBCB) that includes objective, demand, and stress burden subscales (subscales *α* = 0.88, 0.74 and 0.84, respectively) 32, 33. Objective burden is defined as interference with the caregiver's private, social and recreational time, and normal daily routine (e.g., restrictions on vacations and trips, amount of time for friends, amount of personal privacy). Demand burden is defined as the degree of strain on caregivers due to feeling that their care recipients are overly demanding (e.g., attempts by care recipient to manipulate caregiver, unreasonable care recipient demands and requests). Stress burden is defined as the emotional strain felt by caregivers due to caregiving tasks (e.g., life tension, anxiety, depression about the care recipient relationship).

### Statistical methods

*t*‐Tests and Pearson's chi‐square were used to examine demographic and baseline patient outcome differences between patients with and patients without a caregiver. Variables with significant differences between groups were included as covariates in survival analyses (i.e., variables significantly associated with having a family caregiver). Cox proportional hazards regression analyses 34 were used to model: (1) the association between caregiver coparticipant presence/absence and survival (with and without adjustment for baseline covariates) and (2) the association between caregivers' QOL, depressed mood, and burden (objective, demand, and stress burden) measured both at baseline and at the last occasion before patients' death and patient survival. Patients with missing covariate data were excluded as needed for each Cox model.

## Results

Table 1 lists caregiver demographic characteristics. The mean age was 59.4 years. Most caregivers were female (78.0%, *n *= 96); White race (92.7%, *n* = 114); Protestant (33.3%, *n* = 41); married or living with a partner (91.9%, *n* = 113); employed full or part time (49.6%, *n* = 61); and were the patient's spouse/partner (75.6%, *n* = 93). The diagnoses of the care recipients were lung (43.1%, *n* = 53), gastrointestinal (25.2%, *n* = 31), genitourinary (8.1%, *n* = 10), breast (8.1%, *n* = 10), hematologic (5.7%, *n* = 7), and other solid tumor cancers (10.6%, *n* = 13).

**Table 1:** Caregiver Demographic Characteristics (*N *= 123)

Characteristic	No.	%
Age, years		
Mean	59.4	
SD	11.7	
Sex		
Female	96	78.0
Male	26	21.1
Missing	1	.8
Race		
White people	114	92.7
Other	5	4.1
Missing/no response	4	3.3
Marital Status		
Married or living with partner	113	91.9
Never Married	4	3.3
Divorced or separated	3	2.4
Widowed	2	1.6
Missing/no response	1	.8
Education		
High school or GED; some college or technical school	70	56.9
≥ College graduate	51	41.5
<High school graduate	1	.8
Missing/no response	1	.8
Employment status		
Full or part time	61	49.6
Retired	35	28.5
Not employed	25	20.3
Missing/no response	1	.8
Religious affiliation		
Protestant	41	33.3
Catholic	36	29.3
Jewish	2	1.6
None	23	18.7
Other	15	12.2
Missing/no response	6	4.9
Relationship to Patient		
Spouse/partner	93	75.6
Sibling	7	5.7
Son or daughter	14	11.4
Parent	7	5.7
Other	1	.8
Missing/no response	1	.8
Primary disease site of patient		
Lung	53	43.1
GI	31	25.2
GU	10	8.1
Breast	10	8.1
Hematologic	7	5.7
Other solid tumor	13	10.6

Table 2 lists patient characteristics and compares groups of patients with (*n* = 123) and without caregivers (*n* = 84). Compared to patients with*out* caregivers, patients with caregivers were older (62.4 vs. 65.7, *P* = 0.02); male (38.1% vs. 62.6%, *P* < 0.01); married or living with a partner (53.6% vs. 73.2%, *P* < 0.01); and were more likely to have a living will or durable power of attorney (34.5% vs. 49.6%, *P* = 0.05). There were no differences in patients' other demographics, Charlson scores, Karnofsky Performance Status (KPS), symptom impact, depressed mood (CES‐D), QOL (FACIT‐Pal), clinical trial enrollment, presence of a do‐not‐resuscitate (DNR) order, or intervention group.

**Table 2:** Patient characteristics

Characteristic	All patients (*N *= 207)		Patients with a caregiver (*N *= 123)		Patients without a caregiver (*N *= 84)		*P* a
	No.	%	No.	%	No.	%	
Age, years							
Mean	64.3		62.4		62.4		0.02
SD	9.9		9.9		9.9		
Male gender	109	52.7	77	62.6	32	38.1	<0.01
Marital Status							
Married	135	65.2	90	73.2	45	53.6	<0.01
Not married	72	34.8	33	26.8	39	46.4	
Education							
<High school graduate	11	5.3	6	4.9	5	6.0	0.92
High school graduate	111	53.6	67	54.5	44	52.4	
College graduate	85	41.1	50	40.7	35	41.7	
Race							
White people	200	96.6	118	96.7	82	97.6	0.31
Black people	1	.5	0	0	1	1.2	
Other	5	2.4	4	3.3	1	1.2	
Missing	1	.5	1	.01	0	0	
Religion							
Catholic	65	31.4	34	27.6	31	36.9	0.41
Protestant	63	30.4	42	34.1	21	25.0	
Jewish	1	.5	1	.8	0	0	
None	44	21.3	25	20.3	19	22.6	
Other	28	13.5	16	13.0	12	14.3	
Missing	6	2.9	5	4.1	1	1.2	
Employment Status							
Employed	49	23.7	27	22.0	22	26.2	0.56
Retired	99	47.8	61	49.6	38	45.2	
Not Employed	58	28.0	35	28.5	23	27.4	
Student	1	.5	0	0	1	1.2	
Medical insurance							
Medicare	104	50.2	64	52.0	40	48.2	0.47
Private/Commercial	71	34.3	42	34.1	29	34.9	
Military	19	9.2	12	9.8	7	8.4	
Medicaid	7	3.4	4	3.3	3	3.6	
Uninsured	5	2.4	1	.8	4	4.8	
Missing	1	.5	0	0	1	1.2	
Ever smoked	145	70.1	87	70.7	58	69.0	0.80
Diagnosis							
Lung	88	42.5	53	43.1	35	41.7	0.68
Gastrointestinal tract	50	24.2	31	25.2	19	22.6	
Breast	23	11.1	10	8.1	13	15.5	
Other solid tumor	20	9.7	12	10.6	8	9.5	
Genitourinary tract	16	7.7	10	8.1	6	7.1	
Hematologic malignancies	10	4.8	7	5.7	3	3.6	
Charlson score	6.3	1.7 (SD)	6.2	1.6 (SD)	6.3	2.0 (SD)	0.65
Karnofsky Performance Status	81.0	10.3 (SD)	81.1	11.0 (SD)	80.8	9.3 (SD)	0.84
FACIT‐Pal (Baseline)	126.2	21.3	126.6	19.7 (SD)	125.6	23.4 (SD)	0.75
CES‐D (Baseline)	14.2	10.1	13.3	8.8 (SD)	15.5	11.6 (SD)	0.14
QUAL‐E, Symptom Impact Subscale (Baseline)	11.7	3.7 (SD)	11.6	3.7 (SD)	11.8	3.6 (SD)	0.61
In a clinical trial at enrollment	27	13.0	16	13.0	11	13.1	0.71
Advance directive in chart at enrollment							
Living will or durable power of attorney	89	43.0	60	49.6	29	34.5	0.05
DNR order	20	9.7	13	11.7	7	8.5	0.63
Early intervention group	104	50.2	61	51.2	42	49.4	0.95

a Fisher's exact or Pearson's chi‐square test for categorical variables and t‐test for continuous variables. DNR, do‐not‐resuscitate.

### Caregiver status and patient survival

**Figure 1:**
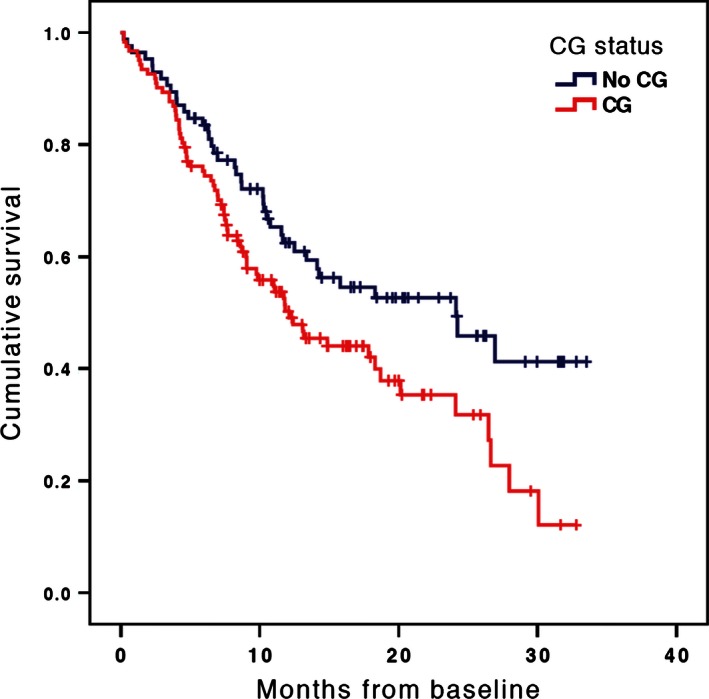
Patient survival curves by caregiver coparticipant presence/absence. Cox proportional hazards model with no covariates.

**Figure 2:**
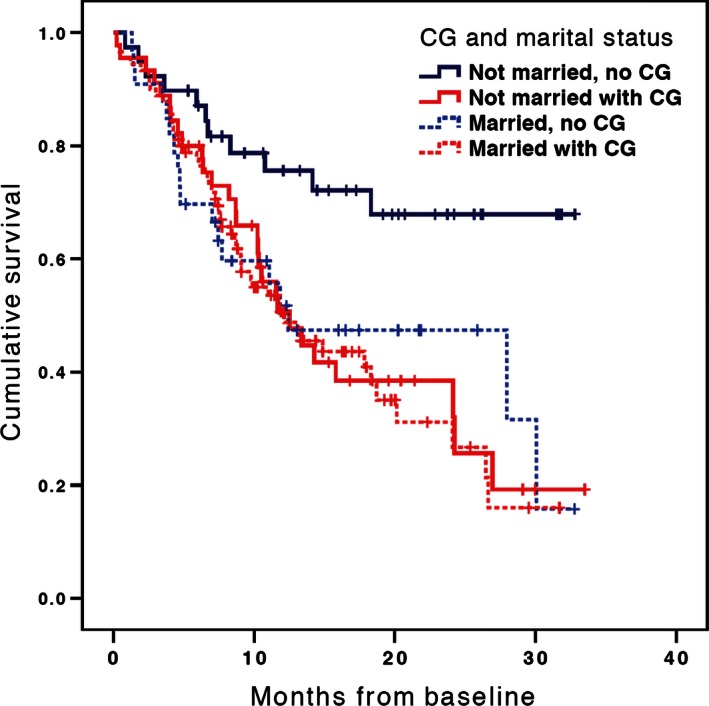
Adjusted survival curves by caregiver and marital status. Cox proportional hazards model adjusted for patient age, gender, and presence of an advance directive and/or durable power of attorney.

In a Cox regression model that included the caregiver status predictor and no covariates, having a caregiver coparticipant was associated with reduced patient survival (*n* = 207, Wald(1) = 4.31, HR = 1.52, CI: 1.02–2.25, *P* = 0.04) (see Fig. 1). In a model that included covariates correlating significantly with caregiver status (patient age, patient gender, marital status [married/unmarried], and advance directive status [see Table 2]), caregiver status was not associated with reduced survival (*n* = 205, Wald(1) = 1.33, HR = 1.28, CI: 0.84–1.96, *P* = 0.25); however, marital status was significantly associated with reduced survival (*n* = 205, Wald(1) = 4.33, HR = 1.62, CI: 1.03–2.56, *P* = 0.04). Because caregiver status was significantly associated with marital status, we conducted a Cox regression analysis that included caregiver status, marital status, and their interaction as simultaneous predictors while covarying patient age, gender, and advance directive status. In this model, caregiver status (*n* = 205, Wald(1) = 6.25, HR = 2.62, CI: 1.23–5.59, *P* = 0.01), marital status (*n* = 205, Wald(1) = 8.79, HR = 2.92, CI: 1.44–5.91, *P* = 0.003), and their interaction (*n* = 205, Wald(1) = 5.18, HR = 0.35, CI: 0.14–0.87, *P* = 0.02) were significant predictors of survival. The interaction took the form such that caregiver status was a significant predictor of survival among unmarried but not among married patients. Hence, when Cox analyses were conducted separately, caregiver status was a significant predictor of reduced survival among unmarried patients (Wald = 5.19, HR = 2.44, CI: 1.13–5.25, *P* = 0.02), but not among married patients (Wald = 0.37, HR = 0.86, CI: 0.52–1.42, *P* = 0.55). In order to illustrate this effect, a fourfold categorical variable was created (caregiver present/married, caregiver present/unmarried, caregiver absent/married, caregiver absent/unmarried). When this variable was entered in a Cox regression model covarying patient age, gender, and advance directive status, it was a significant predictor of patient survival (Wald = 8.79, *P* = 0.02). Kaplan–Meier survival curves were generated for these four groups (see Fig. 2). Unmarried patients without a caregiver (indicated by the solid blue line) experienced better survival than the other three groups.

### Caregiver QOL, depressed mood, and burden and patient survival

**Figure 3:**
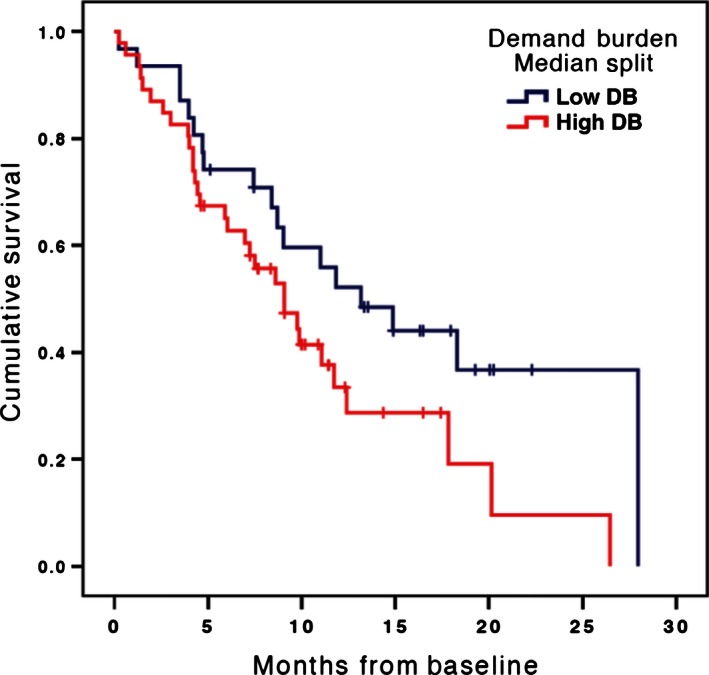
Adjusted survival curves by high and low caregiver burden using median split. Cox proportional hazards model adjusted for intervention group.

At baseline, caregiver QOL, depressed mood, and burden were not predictive of patient survival. At the last measurement period before death, only caregiver demand burden (*n* = 77, Wald(1) = 4.87, CI: 1.01–1.20, *P* = 0.03) (Fig. 3) was significantly related to decreased survival. Caregiver QOL (*n* = 93, Wald(1) = 1.14, CI: 0.99–1.02, *P* = 0.29), depressed mood (*n* = 93, Wald(1) = 1.22, CI: 0.99–1.04, *P* = 0.27), objective (*n* = 81, Wald(1) = 1.68, CI: 0.97–1.15, *P* = 0.20) and stress burden (*n* = 91, Wald(1) = 1.77, CI: 0.97–1.16, *P* = 0.18) were not significant.

### Post Hoc analysis

In our RCT, we collected two measures of a patients' social support that could be related to having a caregiver: the Multidimensional Scale of Perceived Social Support 35 and the Social Well‐being subscale of the FACIT‐Pal 36. Although both measures were related to caregiver status such that patients with caregivers had higher ratings of social support, neither measure was associated with patient survival (both with and without covariates). When these social support measures were included as covariates along with gender, age, and advance directive status, the results for caregiver and marital status remained essentially unchanged as shown in these results: caregiver status (*n* = 203, Wald(1) = 6.90, HR = 2.89, CI: 1.31–6.40, *P* = 0.009), marital status (*n* = 203, Wald(1) = 9.10, HR = 3.11, CI: 1.49–6.49, *P* = 0.003), and their interaction (*n* = 203, Wald(1) = 5.62, HR = 0.33, CI: 0.13–0.83, *P* = 0.02).

### Comment

We conducted a secondary analysis of the ENABLE III RCT data to examine whether advanced cancer patients' higher survival would be associated with having a family caregiver, higher caregiver quality‐of‐life (QOL), and lower caregiver depression. Contrary to our hypothesis, patients with a caregiver coparticipant had lower survival compared to those without. This finding was mostly attributable to the higher survival of unmarried patients without caregiver coparticipants. Of interest, higher caregiver demand burden was associated with lower survival, while caregiver QOL, depression, objective burden, and stress burden were not. This is the first study to show a significant relationship between the survival of advanced cancer patients and family caregivers' presence and burden.

It is unclear why patients with a caregiver had shorter survival compared with those patients in the trial who did not. We offer two possible explanations. First, patients who had a high‐disease burden might have had more daily health needs that required the presence and assistance of a family caregiver. This high‐disease burden might itself be associated with shorter survival; hence having a caregiver might represent sicker patients with a poorer prognosis. However, the available data we collected of potential markers of disease severity did not support this explanation. We found no detectable differences between those with and without a caregiver in baseline cancer diagnoses, Charlson scores, KPS scores, symptom impact, depressed mood, QOL, or clinical trial enrollment (see Table 2). It could be that there are other markers of disease severity that would better predict patient survival and needing a family caregiver. Thus, we recommend that this be explored in future studies.

A second potential explanation for our results could relate to patients' self‐perceived burden on their family caregivers 37, 38, 39, 40. When debilitating illnesses, such as cancer, constrain a patients' ability to care for themselves thereby necessitating assistance from family members, it is possible that these patients begin to see themselves as an undue burden on others. There is some evidence to suggest that this reluctance to burden others may result in depression 40 and impact one's health behaviors and preferences for treatment. For example, a study by Lee and colleagues 38 of 326 patient‐caregiver dyads with distant stage cancers found that both higher patient self‐perceived burden and higher caregiver burden scores were associated with lower preferences for life‐sustaining treatments. While evidence is lacking in our study to directly support the mechanism of a patient's reluctance to burden others, it is notable that compared to patients without caregivers, a higher proportion of patients with caregivers had a living will or durable power of attorney (see Table 2).

When conducting these analyses, we found it puzzling that just over half of the patient samples with no caregiver coparticipants were married (54%). This appears to challenge the adequacy of caregiver coparticipation as a proxy variable for caregiver status since it is reasonable to assume that these married patients may have been receiving some kind of assistance from their spouse. To clarify this puzzle, we included marital status in the Cox regression analysis as a predictor in the 4‐group Kaplan–Meier curves (Fig. 2). This analysis revealed two important insights. First, being married was highly associated with having a caregiver coparticipant (Table 2). Second, the addition of marital status along with caregiver status as predictor variables in the Cox regression showed that the significant association with survival was maintained in the same negative direction, such that having a spouse was also associated with *lower* survival. This is consistent with the 4‐group Kaplan–Meier curves demonstrating that those patients who were unmarried with no caregiver coparticipant had the better survival in comparison to everyone else who was either married and/or had a caregiver coparticipant. Our interpretation of this is that those 54% of married patients with no caregiver coparticipant did in reality have a spouse who may have been providing support, however, this continued to be associated with decreased survival.

We propose several explanations for these findings. First, while numerous studies report a protective effect of marriage on cancer mortality 41, 42, 43, 44, 45, 46, 47, other studies find marriage associated with lower survival 48 (which is consistent with our findings). Alternatively, the relationship may vary depending on the history of a person's marital status: for example, in a meta‐analysis of the association of social networks with cancer mortality 49, survival was lowest for individuals who were *never* married compared to individuals who were divorced, separated or widowed. In our analyses, marital status was operationalized as married/unmarried and hence it is likely that our unmarried patient group reflects a sample that is heterogeneous with regard to marital status history, making it difficult to interpret how marital status drives the findings.

Second, despite controlling for gender in our analyses, unmeasured factors associated with gender may explain our strong study results. Lending support for this hypothesis, a meta‐analysis of 1,365 nonsmall cell lung cancer patients by Siddiqui and colleagues 50 found that unmarried females had higher overall survival than both married and unmarried males. This is especially compelling given that in our study, patients without a caregiver were largely unmarried and female.

Finally, our measure of marital status does not necessarily indicate the quality of patients' relationships and social support networks. A substantial body of literature has shown that the quality of relationships and social support have a significant impact on patients' health and survival 51, 52. In post hoc analyses, social support did correlate with having a caregiver coparticipant. However, neither the MSPSS nor the FACIT‐Pal social well‐being subscale was related to patient survival. Furthermore, inclusion of these social support measures did not change the results of the final model. This suggests that while the constructs of “social support” and “family caregiver” have related features, there are dimensions of having a family caregiver not related to social support that are associated with patient survival. Future research should examine how different types of social support, including the unique type of social support delivered by family caregivers, influence patients' health and longevity.

For patients with a family caregiver, lower survival was associated with higher caregiver demand burden. That is, the patients of caregivers who perceived care recipients and their care to be overly demanding had a higher risk of death. Mirroring the first explanation above, patients with more severe and progressive life‐limiting illness may have a greater need for assistance from caregivers that would impact a caregiver's normal daily routine and sense of a patient's overly demanding situation; however, we found no differences in these patients' markers of disease severity at baseline.

While provocative, these findings are subject to several limitations. First, this was not a prospective, planned analysis. Second, as is common across studies of the seriously ill, we experienced significant caregiver attrition (32%) that could have resulted in a selection bias. It is reasonable to conjecture that those caregivers experiencing higher burden discontinued the study; however, as reported in the trial's primary paper 2, we found no significant associations between attrition and caregiver demographics and outcomes. Third, this study included few caregivers and patients of a minority racial group, whose burden has been shown to differ from Whites 53, thus limiting generalizability. Fourth, it is not entirely known what proportion of patients who did not elect to have a caregiver participate did in fact have close family and friends who assisted them in some way with their care. In other words, patients with or without caregiver co‐participants may not equate with actual caregivers available to patients. Future studies will benefit from having more detailed information about family support regardless of whether patients elect a caregiver to co‐participate.

To conclude, we believe the results of this analysis are surprising relative to current literature and raise more questions than are answered. However, these findings are important as they serve to challenge our assumptions about the impact of family caregiving on patient outcomes. We echo what the Institute of Medicine 6, 54 and others 16, 55 have recently emphasized, namely that there is a critical need to prioritize and place special emphasis on research and interventions aimed at better understanding and supporting family caregiving for the critically ill and dying. The scope of this need is vast, as most of the over half million individuals with advanced cancer who are in their last year of life 56 have a family member or close friend who assists them on a daily basis. Not only are these family caregivers encumbered with delivering nearly all of a patient's daily care, they are burdened with witnessing someone close to them struggle with life‐limiting illness. Hence, it is imperative that palliative and oncology clinicians' work together to ensure that these caregivers are supported in their role. Understanding how to best provide this support will be greatly benefited by continuing to improve our understanding of the complex dynamic between seriously ill patients and their family caregivers.

## Conflict of Interest

The authors have no conflict of interests to disclose.
